# Human Milk Oligosaccharides: 2′-Fucosyllactose (2′-FL) and Lacto-N-Neotetraose (LNnT) in Infant Formula

**DOI:** 10.3390/nu10091161

**Published:** 2018-08-24

**Authors:** Yvan Vandenplas, Bernard Berger, Virgilio Paolo Carnielli, Janusz Ksiazyk, Hanna Lagström, Manuel Sanchez Luna, Natalia Migacheva, Jean-Marc Mosselmans, Jean-Charles Picaud, Mike Possner, Atul Singhal, Martin Wabitsch

**Affiliations:** 1 KidZ Health Castle, UZ Brussel, Vrije Universiteit Brussel, 1090 Brussels, Belgium; 2 Department of Gastro-Intestinal Health, Nestlé Institute of Health Sciences, Nestlé Research, Nestec Ltd., 1015 Lausanne, Switzerland; bernard.berger@rdls.nestle.com; 3 Neonatal Pediatrics, Polytechnic University of Marche, 60121 Ancona, Italy; v.carnielli@univpm.it; 4 Department of Paediatrics, Nutrition and Metabolic Diseases, The Children’s Memorial Health Institute, 04-730 Warsaw, Poland; J.Ksiazyk@IPCZD.Pl; 5 Department of Public Health, University of Turku and Turku University Hospital, 20014 Turku, Finland; hanlag@utu.fi; 6 Neonatology Division, Research Institute University Hospital Gregorio Marañón, Complutense University, 28009 Madrid, Spain; msluna@salud.madrid.org; 7 Department of Pediatrics, Samara State Medical University, 443084 Samara, Russia; nbmigacheva@gmail.com; 8 Citadelle For Life, 1000 Brussels, Belgium; jmm.mosselmans@gmail.com; 9 Neonatology, Croix-Rousse Hospital, Lyon and CarMen Unit, INSERM U1060, INRA U197, Claude Bernard University, 69100 Lyon 1, France; jean-charles.picaud@chu-lyon.fr; 10 Nestlé Nutrition Institute, 60528 Frankfurt/Main, Germany; Mike.Possner@de.nestle.com; 11 Paediatric Nutrition, UCL Great Ormond Street Institute of Child Health, London WC1N 1EH, UK; a.singhal@ucl.ac.uk; 12 Department of Paediatrics and Adolescent Medicine, Division of Paediatric Endocrinology and Diabetes, Centre for Hormonal Disorders in Children and Adolescents, Ulm University Hospital, 89075 Ulm, Germany; Martin.Wabitsch@uniklinik-ulm.de

**Keywords:** breast feeding, formula feeding, human milk oligosaccharide, 2′-fucosyllactose, Lacto-N-neotetraose, microbiota, bifidobacteria

## Abstract

The authors reviewed the published evidence on the presence of oligosaccharides in human milk (HMO) and their benefits in in vitro and in vivo studies. The still limited data of trials evaluating the effect of mainly 2′-fucosyllactose (2′-FL) on the addition of some of HMOs to infant formula were also reviewed. PubMed was searched from January 1990 to April 2018. The amount of HMOs in mother’s milk is a dynamic process as it changes over time. Many factors, such as duration of lactation, environmental, and genetic factors, influence the amount of HMOs. HMOs may support immune function development and provide protection against infectious diseases directly through the interaction of the gut epithelial cells or indirectly through the modulation of the gut microbiota, including the stimulation of the bifidobacteria. The limited clinical data suggest that the addition of HMOs to infant formula seems to be safe and well tolerated, inducing a normal growth and suggesting a trend towards health benefits. HMOs are one of the major differences between cow’s milk and human milk, and available evidence indicates that these components do have a health promoting benefit. The addition of one or two of these components to infant formula is safe, and brings infant formula closer to human milk. More prospective, randomized trials in infants are need to evaluate the clinical benefit of supplementing infant formula with HMOs.

## 1. Introduction

Breast milk is the natural and ideal food for infants, providing the energy and nutrients that every infant needs during the first four to six months of life in the correct quality and amount. Infants who are breastfed for shorter periods or are not breastfed suffer more infectious diseases, such as gastroenteritis and acute otitis media, more immune-mediated diseases, have a lower intelligent quotient (IQ) and are likely to have a higher risk of being overweight and type 2 diabetes in later life [1,2]. However, any breastfeeding is beneficial. In a pooled analysis of 24 studies from the USA and Europe, for example, any form of breastfeeding was found to be protective for acute otitis media in the first two years of life, but exclusive breastfeeding for the first six months was associated with the greatest protection [3].

The composition of breast milk is unique. Aside from nutrients for the infant’s healthy growth and development, it contains thousands of bioactive substances [4], including human milk oligosaccharides (HMOs) [5]. HMOs are non-digestible carbohydrates [6]. Although they have little nutritional value for the infant, HMOs are the third largest solid component in human milk after lactose and lipids [7,8]. More than 200 free oligosaccharide structures have so far been identified from human milk samples [9]. Compared to human milk, oligosaccharide concentrations in the milk of farm animals, such as cows, goats, and sheep are 100–1000-fold lower. In fact, these unique complex carbohydrate structures in human milk are virtually absent in cow’s milk or any other farmed animal milk, and their variety is much lower [10]. The difference in oligosaccharide content on human milk and cow milk, and, thus, cow milk-based infant formula, is likely to explain, at least in part, the differences in health outcomes between formula and breastfed infants.

## 2. Human Milk Oligosaccharides 

Around 1900, infant mortality rate (deaths in the first year of life per 1000 live births) in Europe was very high at up to and above 20% [11,12]. Mortality was especially high in non-breastfed infants and was seven times greater in bottle-fed than breastfed infants [11,13]. It was around this time that differences in stool bacterial composition were discovered between breastfed and formula-fed infants and both breastfeeding and the resulting gut microbiota were linked to the better health of the infants [11,13]. In the 1930s, oligosaccharides were identified as the most important bifidogenic factor in human milk [11]. The most abundant oligosaccharides in human milk were discovered in 1954. However, it was only recently that scientists and industry were able to produce the first oligosaccharides structurally identical to those in human milk [14]. It is important to note that HMOs resist cold and heat and are not affected by pasteurization and freeze-drying [15,16].

The amount of HMOs is 20–25 g/L in colostrum and 10–15 gram per liter (g/L) in mature milk, or 1.5–2.3 g/100 kcal assuming an energy density of human milk of 64 kcal/100 mL [13,17]. Three major HMO categories are present in breast milk: (i) fucosylated neutral HMOs (35–50%); (ii) sialylated acidic HMOs (12–14%), and (iii) non-fucosylated neutral HMOs (42–55%) [18,19]. Neutral HMOs account for more than 75% of the total HMOs in human breast milk. 2′-fucosyllactose (2′-FL) is part of the fucosylated, while Lacto-N-neotetraose (LNnT) is part of the non-fucosylated neutral HMOs. In women who are “secretors”, 2′-FL is by far the most abundant HMO and constitutes nearly 30% of all HMOs.

All HMOs are synthesized in the mammary gland [20]. The amount and composition of HMOs vary between women and over the course of lactation. HMO concentration is higher during the early stages of lactation and decreases gradually over time [21,22,23]. The Lewis antigen system is a human blood group system based upon two genes on chromosome 19: fucosyltransferase-3 (*FUT3)*, or Lewis gene; and *FUT2*, or Secretor gene. *FUT2* has a dominant allele which codes for an enzyme and a recessive allele which does not produce a functional enzyme. Similarly, *FUT3* has a functional dominant allele and a non-functional recessive allele. A recent study could not confirm the observation that the content of HMOs is also higher after a term than preterm delivery [24]. The most extreme intra individual variation in HMO fucosylation is based on the maternal secretor and Lewis blood group status [13,20,24]. Both the *FUT2* or secretor gene and the *FUT3* or Lewis gene are expressed in glandular epithelia. The secretor (*Se*) gene encodes for the *FUT2* which is necessary for the synthesis of 2′-FL and other Fucosyl-HMOs and is expressed in the lactating mammary gland. The milk of secretor (*Se*+) women is, therefore, characterized by an abundance of α1-2-fucosylated HMOs, especially 2′-FL [6,13,24]. Non-secretors, by contrast, lack the *FUT2* enzyme and, therefore, their milk does not contain 2′-FL and other α1-2-fucosylated HMOs, or is in only minimal amounts [6,25]. The absence of 2′-FL and other α1-2-fucosylated HMOs explains the lower total amount of HMOs in “non-secretor” milk [20]. For example, a recent study found approximately 35% to 45% less total HMOs in the milk of non-secretor Lewis-positive women than in the milk of Lewis-positive secretor women [20]. The acidic HMOs do not depend on secretor status [20].

Based on the expression of *FUT2* and *FUT3*, breast milk can be assigned to one of four groups (Table 1) [13]:

Group 1: Secretors, Lewis-positive, (Se+Le+) (*FUT2* active, *FUT3* active)
Group 2: Non-secretors, Lewis-positive, (Se−Le+) (*FUT2* inactive, *FUT3* active)
Group 3: Secretors, Lewis-negative (Se+Le−) (*FUT2* active, *FUT3* inactive)
Group 4: Non-secretors, Lewis-negative (Se−Le−) (*FUT2* inactive, *FUT3* inactive)

About 80% of the European and American women are secretors [26]. About 70% of the populations are Lewis-positive secretors (Se+Le+) and around 5–10% are Lewis-negative secretors (Se+Le−) [27].

However, other factors also influence HMO synthesis. A recent study showed that HMO content and profiles vary geographically, even when secretor and Lewis blood group genes were considered [20,28]. Findings on HMO concentrations over time of lactation and clusters bas ed on 2′-FL concentrations suggest that LNnT and Lacto-N-Tetraose (LNT) are ‘co-regulated’ with the FUT2 dependent 2′-FL concentration, with LNnT showing a positive and LNT a negative relation to the amount of 2′-FL [6]. Mothers’ milk with low levels of 2′-FL also contains low levels of LNnT but high levels of LNT [6]. The clinical impact of these findings still needs to be unraveled.

The European Union (EU) considers two HMOs, 2′-FL and LNnT, novel foods (Commission Implemented Regulation (EU) 2017/2470). Today, the USA’s FDA considers three HMOs to be Generally Regarded as Safe (GRAS notice no 650). On 29 June 2015, the European Food Safety Authority (EFSA), based on the scientific and technical information provided, concluded that 2′-FL is safe for infants up to one year of age when added to infant and follow-on formulae, in combination with LNnT, at concentrations up to 1.2 g/L of 2′-FL and up to 0.6 g/L of LNnT, at a ratio of 2:1 in the reconstituted formulae. 2′-FL is safe for young children (older than one year of age) when added to follow-on and young-child formulae, at concentrations up to 1.2 g/L of 2′-FL (alone or in combination with LNnT, at concentrations up to 0.6 g/L, at a ratio of 2:1) (EFSA-Q-2015-00052, EFSA Journal 2015).

## 3. Health Benefit of Human Milk Oligosaccharides

Secretor milk (due to its high levels of 2′-FL and other Fucosyl-HMOs) may have advantages for the infant because it more effectively promotes an early high Bifidobacteria-dominated gut microbiota [29] and provides better protection against specific diarrheal diseases [30] than non-secretor milk.

Several studies have documented beneficial effects of HMOs, including modification of the intestinal microbiota, anti-adhesive antimicrobial effects, modulation of intestinal epithelial cell response, effects on immune development and on brain development.

## 4. Preclinical and Observational Studies

### 4.1. Modification of the Intestinal Microbiota

In vitro studies have shown that HMOs promote the growth of certain, but not all, bifidobacteria [13]. *Bifidobacterium longum subsp. infantis (B. infantis)* grows well on HMOs (including 2′-FL) as the sole source of carbohydrates [31,32,33,34]. Compared to *B. infantis*, *Bifidobacterium bifidum* grows slightly slower on HMOs [33]. Recent literature showed in strains from other bifidobacterial species that the metabolic capacity to utilize HMOs is not restricted to *B. infantis* [35,36,37].

Observational studies showed that 2′-Fucosyl-HMOs are associated with bifidobacteria dominated early gut microbiota in breastfed infants [29,35,38].

The fact that HMOs are a preferred substrate for *B. infantis* and other bifidobacteria strains may reduce the nutrients available for potentially harmful bacteria and keep their growth under control. In addition, *B. infantis* produces short-chain fatty acids (SCFA), which help create an environment favoring the growth of commensal bacteria instead of potential pathogens [39].

An in vitro study evaluated HMOs’ utilization by *Enterobacteriaceae*, which has been linked to the onset of necrotizing enterocolitis (NEC) in preterm infants [40]. The study showed that none of the *Enterobacteriaceae* strains grow on 2′-FL, 6-siallylactose (6′-SL), and LNnT, whereas several *Enterobacteriacea* strains, including pathogens, grew well on galacto-oligosaccharides (GOS) [40]. The influence of secretor status and breastfeeding on gut microbiota composition persists up to two to three years [38].

### 4.2. Anti-Adhesive Antimicrobial

Many viruses, bacterial pathogens or toxins need to adhere to mucosal surfaces to colonize or invade the host and cause disease [13,41,42]. Some HMOs are structurally similar to the intestinal epithelial cell surface glycan receptors and serve as decoy receptors to prevent pathogen binding and enhance pathogen clearance [13]. This unique beneficial effect of HMOs is highly dependent on their structure.

Evidence for an anti-adhesive effect of specific HMOs comes from in vitro and ex vivo studies. For instance, Ruiz-Palacios et al. demonstrated that human milk oligosaccharides inhibited *Campylobacter jejuni (C. jejuni)* adherence to epithelial cells in vitro [43], one of the major causes of bacterial diarrhea worldwide. A second study conducted by the same group confirmed that fucosylated human milk oligosaccharides inhibit *Campylobacter* colonization of human intestinal mucosa ex vivo [43]. Yu et al. tested the ability of 2′-FL to inhibit *C. jejuni* infection of the intestinal epithelium and *C. jejuni*-associated mucosal inflammation [44]. In an in vitro model, 2′-FL attenuated 80% of *C. jejuni* invasion (*p* < 0.05) and decreased the release of mucosal pro-inflammatory signals. In a mouse model, ingestion of 2′-FL reduced *C. jejuni* colonization by 80%, weight loss by 5%, intestinal inflammation (shown by histologic features), and induction of inflammatory signaling molecules (*p* < 0.05) [44].

In infants, observations from a prospective study conducted by Morrow et al. suggested a beneficial effect of α1-2-fucosylated HMO on reducing episodes of *C. jejuni*-associated diarrhea [30]. In Mexican breastfed infants, *Campylobacter* diarrhea occurred less often in those infants whose mother’s milk contained a high percentage of milk oligosaccharides of 2′-FL than in those infant whose mother’s milk contained a lower percentage of 2′-FL oligosaccharides. There was a dose-dependent association with higher rates of moderate-to-severe diarrhea of all causes. The association between milk oligosaccharides measured during the first months and diarrhea in breastfed infants persisted through the course of lactation but not after cessation of breastfeeding [30].

Other observational studies of breastfed infants also suggested beneficial effects of fucosyl-HMOs in breast milk. They showed that fucosyl-HMOs in breast milk are related to lower morbidity in Gambian infants at four months of age [45] and fewer respiratory and enteric problems in US infants at three months of age [46].

The influence of 2′-FL and 6′-SL on adhesion of *Escherichia coli* and *Salmonella fyris* to Caco-2 cells was tested with positive results for *E. coli* but not for *Salmonella* [47].

HMOs have also been suggested to possibly protect against important systemic infections of the newborn. For instance, LNnT reduces *Streptococcus (S.) pneumoniae* load in lungs in a rabbit model [48]. A clinical study with infants older than six months, however, could not achieve a reduction in the colonization of the oropharynx with *S. pneumoniae* through a synthetic LNnT-supplemented infant formula [49].

HMOs may function as an alternative substrate to modify a group B *Streptococcus* component in a manner that impairs growth kinetics [50]. There is a unique antibacterial role for HMOs against this leading neonatal pathogen [50].

There is increasing evidence that HMOs could reduce infant mortality and morbidity in preterm infants, for example by shaping a favorable gut microbiome protecting against NEC, candidiasis, and several other immune-related diseases [51]. In support of this, a lower concentration of the HMO disialyllacto-N-tetraose (DSNLT) was shown to predict the risk of NEC in preterm infants. This finding has been demonstrated by a recent multicenter clinical cohort study including 200 mothers and their very low birthweight infants who were predominantly human milk-fed [52]. DSLNT concentrations were significantly lower in almost all milk samples in NEC cases compared with controls, and its abundance could identify NEC cases before onset, i.e., DSLNT content in breast milk is a potential non-invasive marker to identify infants at risk of developing NEC, and screen high-risk donor milk. Beneficial effects on NEC have also been reported for 2′-FL. Good et al. demonstrated that 2′-FL attenuates the severity of the experimental NEC by enhancing mesenteric perfusion in the neonatal intestine on an experimental mouse model of NEC [53].

### 4.3. Modulators of Intestinal Epithelial Cell Response

HMOs are able to reduce cell growth, induce differentiation, apoptosis and maturation, and increase the barrier function in vitro [54,55,56,57]. Intestinal health and intestinal barrier function constitute the first defense line in innate immunity.

Zehra et al. demonstrated that the HMOs 6′-siallyllactose and 2′-FL modulate human epithelial cell responses related to allergic disease in different ways [58]. 6′-Sialyllactose inhibited chemokine (Interleukin (IL)-8 and CCL20) release from T-84 and HT-29 cells stimulated with antigen-antibody complex tumor necrosis factor-alfa (TNF-α) or prostaglandin-E2 (PGE-2); an effect that was PPARy dependent and associated with decreased activity of the transcription factors AP-1 and nuclear factor kappa-light-chain-enhancer of activated B cells) NF-κB. In contrast, 2′-FL selectively inhibited CCL20 release in response to the antigen-antibody complex in PPARy dependent manner. These findings reinforce the concept that structurally different oligosaccharides have distinct biological activities and identifies, and for the first time, that the HMOs, 6′-SL, and 2′-FL, modulate human epithelial cell responses related to allergic diseases. This encourages further investigation of the therapeutic potential of specific HMOs in food allergy [58].

### 4.4. Immune Modulators

Among the multiple functions of HMOs, immunomodulation is one of the most remarkable [59]. HMOs directly affect intestinal epithelial cells and modulate their gene expression, which leads to changes in cell surface glycans and other cell responses. HMOs modulate lymphocyte cytokine production, potentially leading to a more balanced TH1/TH2 response.

An increasing number of in vitro studies suggest that HMOs not only affect the infant’s immune system indirectly by changing gut microbiota but also directly modulate immune responses by affecting immune cell populations and cytokine secretion [5]. HMOs may either act locally on cells of the mucosa-associated lymphoid tissues or on a systemic level [13].

Dietary HMOs were more effective than non-human prebiotic oligosaccharides in altering systemic and gastrointestinal immune cells in pigs [60]. These altered immune cell populations may mediate the effects of dietary HMOs on rotavirus infection susceptibility [60]. Daily oral treatment with 2′-FL attenuated food allergy symptoms in a mouth model by induction of IL-10+ T-regulatory cells and indirect stabilization of mast cells [61].

In vitro studies have shown that 2′-FL directly inhibits lypopolysaccharide-mediated inflammation during enterotoxigenic *E. coli* (ETEC) invasion of T84 (modeling mature) and H4 (modeling immature) intestinal epithelial cells through attenuation of CD14 induction [62]. CD14 expression mediates lypoplysaccharide-TLR4 (toll-like receptor 4) stimulation of portions of the ‘macrophage migration inhibitory factors’ inflammatory pathway via suppressors of cytokine signaling 2/signal transducer and activator of transcription 3/NF-κB.

In an animal model, early life provision for a period of 6 weeks of 1% authentic HMOs delayed and suppressed Type 1 diabetes development in non-obese diabetic mice and reduced the development of severe pancreatic insulitis in later life [63]. In a murine influenza vaccination model dietary 2′-FL improved both humoral and cellular immune responses to vaccination in mice, enhancing vaccine specific delayed-type hypersensitivity responses accompanied by increased serum levels of vaccine-specific immunoglobulin proliferation. Vaccine-specific CD4+ and CD8+ T-cells, as well as interferon-gamma production, were significantly increased in spleen cells of mice receiving 2′-FL leading to the conclusion that dietary intervention with 2′-FL improved both humoral and cellular immune responses to vaccination in mice [64].

### 4.5. Brain Development

Metabolic products of HMOs such as sialic acid promote brain development, neuronal transmission, and synaptogenesis. HMOs provide sialic acid as potentially essential nutrients for brain development and cognition [65,66]. Application of L-fucose and 2′-FL increases the potentiation of the population spike amplitude (POP-spike) and the field excitatory postsynaptic potential (fEPSP) after tetanization of the Schaffer collaterals of the rat hippocampus [67]. Dietary 2′-FL interferes with cognitive domains and improves learning and memory in rodents [68]. HMOs, 3′-Sialyllactose and 6′-Sialyllactose, support normal microbial communities and behavioral responses during stressor exposure, potentially through effects on the gut microbiota–brain axis [69].

### 4.6. Improved Gut Adaptation after Resection

Patients with short bowel syndrome require parental nutrition and may require frequent treatment with antibiotics that modify intestinal microbiota and have an adverse effect on gastrointestinal function [70]. The hypothesis that 2′-FL contributes to the adaptive response after intestinal resection was confirmed on the basis of a murine model of intestinal adaptation. Modulating of gut microbiota following intestinal resection improved the outcome of short bowel syndrome in an experimental setting. Supplementation with 2′-FL increased weight gain following ileo-cecal resection and promoted histological changes in gut mucosa suitable for adaptation [71].

## 5. Clinical Studies with 2′-fucosyllactose

One prospective, randomized, controlled study tested the tolerance and safety in relation to growth of an infant formula containing 2′-FL (0.2 g/L or 1.0 g/L) in combination with galacto-oligosaccahrides (2.2 g/L or 1.4 g/L) in healthy full-term infants from 28 sites across USA [72]. No differences in growth parameters and adverse events were found between the infants fed the formula with 2′-FL from enrolment (0–5 days of age) up to the age of four months compared to infants fed a control formula containing only galacto-oligosaccharides and between both formula-fed groups and the breastfed reference group. This study was the first publication showing that growth of infants consuming a formula containing 2′-FL was similar to that of human milk-fed infants [72]. Both formulas with 2′-FL and galacto-oligosaccharides were well tolerated and did not influence stool frequency or consistency.

The effects of feeding formulas supplemented with 2′-FL on biomarkers of immune function were investigated in a subgroup of this study population [73]. Infants fed formulas with 2′-FL and galacto-oligosaccharides had 29–83% lower concentrations of plasma inflammatory cytokines and TNF-α than infants fed the control formula with galacto-oligosaccharides only [73]. There were no differences in plasma inflammatory cytokines and TNF-α between infants fed formulas with 2′-FL and galacto-oligosaccharides and infants breastfed. These findings indicate that supplementation of infant formula with 2′-FL supports aspects of immune development and regulation similar to that of breastfed infants; while supplementation with galacto-oligosachairdes alone does not [73].

Another prospective, randomized, controlled study tested the gastrointestinal tolerance of an infant formula containing 2′-FL (0.2 g/L) in combination with fructo-oligosaccharides (2 g/L) compared to a control formula without oligosaccharides [74]. The formula with 2′-FL and fructo-oligosaccharides fed from less than eight days of age for approximately one month was well tolerated; stool consistency, anthropometric data, and frequency of feedings with spitting up/vomiting and was similar to that of infants given formula without oligosaccharides or to infants breastfed [74].

Puccio et al. conducted the first clinical trial with an infant formula supplemented with two HMOs [75]. In this prospective, randomized, controlled multicenter study, healthy term infants received a formula with 2′-FL and LNnT or the same formula without HMOs from enrolment at ≤14 days of age to age six months and for at least four months as the exclusive diet [75]. The formula with 2′-FL and LNnT was well-tolerated and supported age-appropriate growth. Gastrointestinal symptoms (flatulence, spitting up, and vomiting) were similar between the groups. Infants receiving formula with 2′-FL and LNnT had significantly softer stools and fewer episodes of night-time wake-ups at age two months, and infants born by caesarian section also had a lower incidence of colic at four months of age. Puccio et al. also analyzed the incidence of different health outcomes as secondary outcomes. Infants fed the formula with 2′-FL and LNnT compared to infants fed the formula without HMOs had significantly fewer parental reports of bronchitis (at 4, 6, and 12 months), reduced incidence of lower respiratory tract infections (through 12 months), reduced use of antipyretics (through four months) and reduced use of antibiotics (through 6 and 12 months) with protective effects that continued after the six months intervention period [75].

In the same trial, infants fed the formula with 2′-FL and LNnT developed a gut microbiota that was closer to the microbiota observed in breastfed infants [76]. At three months of age, the stool microbiota was characterized by an increased quantity of beneficial bifidobacteria and decreased abundances of taxa with potentially pathogenic members. Moreover, the supplementation of infant formula with these two HMOs promoted the growth of a distinct fecal bacteria community, typical of breastfed infants and showing a very high density of bacteria. Formula-fed infants carrying this fecal community type had a two times decreased risk of requiring antibiotics during the first year of life [76]. Therefore, this study suggests that the association between consuming formula with 2′-FL and LNnT and lower parent-reported morbidity and medication use may be linked to gut microbiota community types [76].

Today, the amount of data available on HMO supplementation in infant formula from clinical trials in infants is still limited. More data are definitely needed. According to the data from the few studies, differences in clinical outcome of supplemented vs. non-supplemented formula are not yet conclusive [72,73,74,75,76]. The different primary outcomes of the different trials contribute to a lack of coherent results. The cost-benefit ratio also needs further evaluation. In addition, the optimal concentration of HMO added needs further adjustment. And of course, there is the fact that only one or two HMOs are added to infant formula, while mother’s milk contains 200 different oligosaccharides. Supplementation with more HMOs could result in further evidence of benefit.

## 6. Conclusions

HMOs act as soluble decoy receptors that block the attachment of specific viral, bacterial or protozoan parasite pathogens to epithelial cell surface sugars, which may, in turn, help prevent infectious diseases in the gut, respiratory, and urinary tracts. In addition, HMOs alter host epithelial and immune cell responses with potential benefits for the neonate, beyond protection against infectious diseases.

Although the functions of HMOs have been known for many years, it was not possible to synthesize them on an industrial scale until recently. With the goal of imitating their effect, non-human milk oligosaccharides, mainly fructo and galacto-oligosaccharides have been added to infant formula. In recent years it has, to a certain extent, become technically possible to add 2′-FL and LNnT to infant formula.

The addition of one HMO, namely 2′-FL, is a step forward in bringing formula feeding closer to the gold standard: Mother’s milk. No adverse effects have been reported for 2′-FL and in vitro and animal studies have shown benefits of supplementation of infant formula with 2′-FL. The first clinical data in infants show a normal growth pattern and normal defecation and suggest clinical benefit. More prospective, randomized trials in infants comparing formula without and with HMOs are still needed to evaluate the clinical effects of this supplementation. It can, therefore, be concluded that 2′-FL is a safe supplementation of infant formula.

## Figures and Tables

**Table 1:** Diversity of human milk oligosaccharides (HMO) based on genetic background of the mother.

Gene	Lewis Gene +	Lewis Gene −
Secretor gene +	Lewis positive secretors	Lewis negative secretors
	Secrete all HMOs	Secrete 2′-FL, 3′-FL, LNFP-I, LNFP-III
Secretor gene −	Lewis positive non-secretors	Lewis negative non-secretors
	Secrete 3′-FL, LNFP-II and LNFP III	Secrete 3′-FL, LNFP-III and LNFP-V

2′-FL: 2′-fucosyllactose; 3′-FL: 3′-fucosyllactose; LNFP: Lacto-N-fucopentaose.
